# Toxic thyroid adenoma with hypercalcemia mimicking an intra-thyroidal parathyroid adenoma

**DOI:** 10.22038/AOJNMB.2021.57103.1398

**Published:** 2022

**Authors:** Venkata Subramanian Krishnaraju, Ritesh Upadhyay, Ashwani Sood, Anish Bhattacharya, Bhagwant Rai Mittal

**Affiliations:** Department of Nuclear Medicine, Postgraduate Institute of Medical Education and Research, Chandigarh, India

**Keywords:** Thyroid adenoma hypercalcemia, Parathyroid adenoma, Sestamibi, Pertechnetate

## Abstract

Hypercalcemia is a clinical condition characterized by elevated circulating serum calcium levels either due to raised parathyroid hormone in hyperparathyroidism or due to secondary causes of hypercalcemia without elevated parathyroid hormone levels. However, hyperthyroidism may occasionally present with incidentally detected hypercalcemia. We present a case of a 53-year-old woman with a previous history of an underlying thyroid disorder, now presented with features of hypercalcemia and mildly elevated parathyroid hormone levels. Her ultrasonography of the neck was suggestive of an intra-thyroidal parathyroid adenoma and it was localized as a tracer avid lesion within the thyroid gland on dual-phase ^99m^Tc-sestamibi planar scintigraphy with single photon emission computed tomography/ computed tomography (SPECT/CT). However, a subsequent thyroid profile followed by ^99m^Tc- pertechnetate thyroid scintigraphy showed a hot nodule in the thyroid gland which changed the diagnosis to a toxic thyroid adenoma. She was treated with radioactive iodine ablation and thyrotoxicosis resolved and the serum calcium levels normalized on her follow-up.

## Introduction

 Toxic adenoma of the thyroid gland is a condition characterized by the presence of autonomous functioning thyroid tissue with elevated levels of T_3_ and T_4_ hormones and suppressed TSH. This results in the features of thyrotoxicosis in the form of tachycardia, weight loss, sweating, and increased appetite in the patients (1, 2); however, hypercalcemia has also been identified as one of the uncommon presentations of thyrotoxicosis (3). Here we present a case of a 53-year-old woman who had a nodular lesion in the thyroid gland with incidentally detected hypercalcemia.

## Case Report

**Figure 1:**
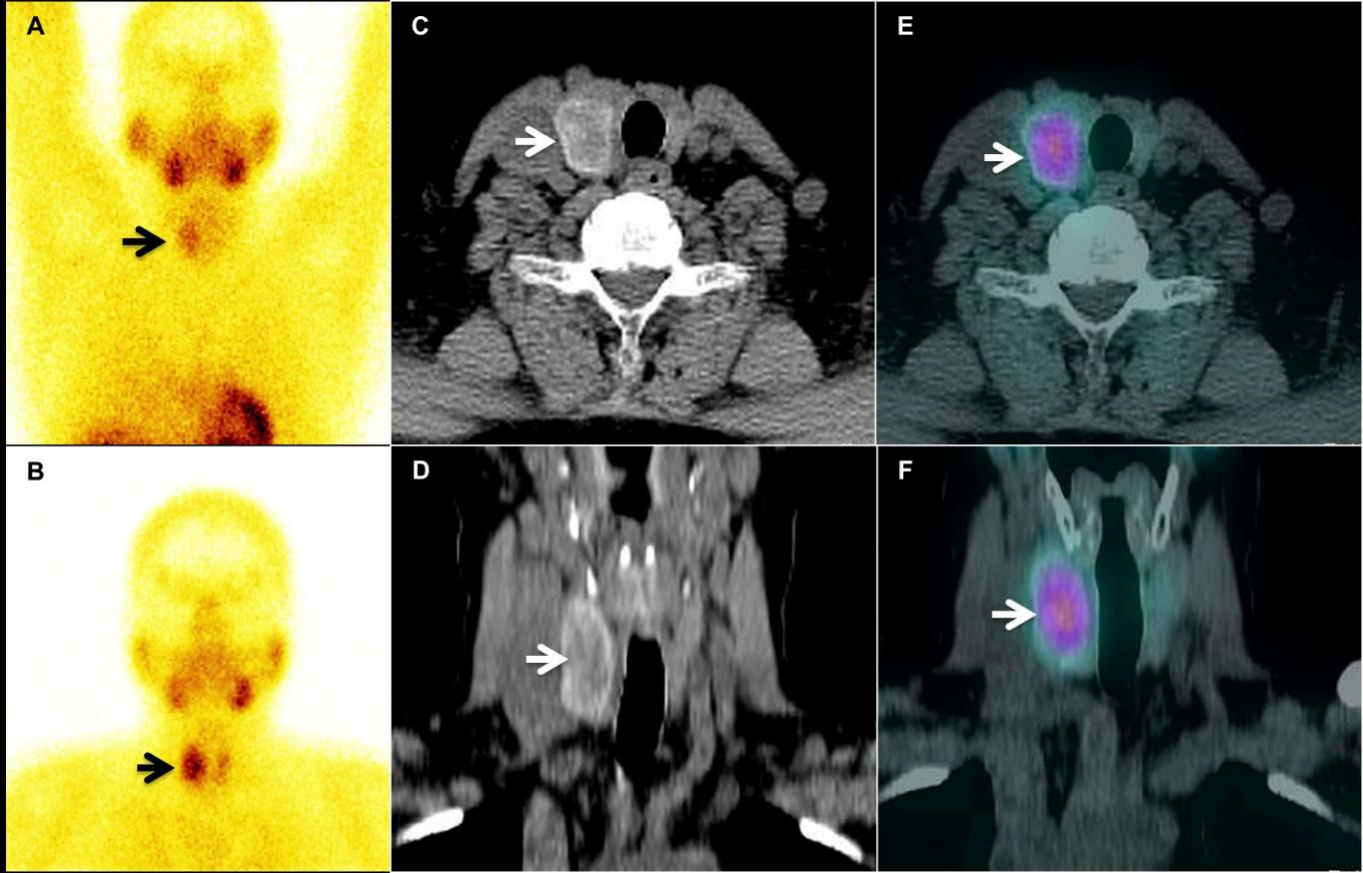
The early-phase planar static image acquired 10 minutes post-injection of 20 mCi (~740 MBq) of Tc-99m sestamibi (**A**) revealed a focus of relatively increased tracer uptake in the right lobe of the thyroid gland with differentially lower washout compared to the rest of the gland on the delayed static image at 1 hour (**B**). The early SPECT/CT images localized the tracer activity to a heterogeneous hypodense lesion within the right lobe of the thyroid gland on the axial and coronal CT (**C,D**) and corresponding fused SPECT/CT images (**E,F**). There were areas of central cystic/necrotic changes within the lesion on the CT images with no other abnormal tracer avid lesion elsewhere in the neck or the mediastinum

 A 53-year-old asymptomatic woman presented with incidentally detected hypercalcemia of 11.8 mg/dl (normal range 8.5-10.3). She had under-lying thyroid disorder with hypothyroidism on thyroid hormone replacement (25 mcg once daily) for the past two years. However, she had discontinued for the last year because of her poor compliance. Her intact parathyroid hormone (iPTH) levels were also mildly elevated (76.7 pg/ml; normal range: 15-65). With suspicion of primary hyperparathyroidism, she was subjected to ultrasonography (USG) of the neck which revealed a heterogeneously hyperechoic nodule of size 2.8×2.1×1.5 cm within the right lobe of the thyroid, having a few small cystic areas within it, suggestive of an intra-thyroidal parathyroid adenoma (PA). There was no other enlarged parathyroid gland(s) found elsewhere in the neck on the USG. Tc-99m sestamibi dual-phase parathyroid planar and single photon emission computed tomography/ computed tomography (SPECT/CT) scintigraphy performed for pre-operative confirmation and localization of additional PA, showed a tracer avid nodule in the right lobe of the thyroid gland with differentially slower washout in the delayed phase (Figure 1). Though the iPTH levels were not significantly elevated as expected in a case of a large parathyroid adenoma, a diagnosis of an intra-thyroidal parathyroid adenoma was made and the patient was planned for surgical excision.

**Figure 2:**
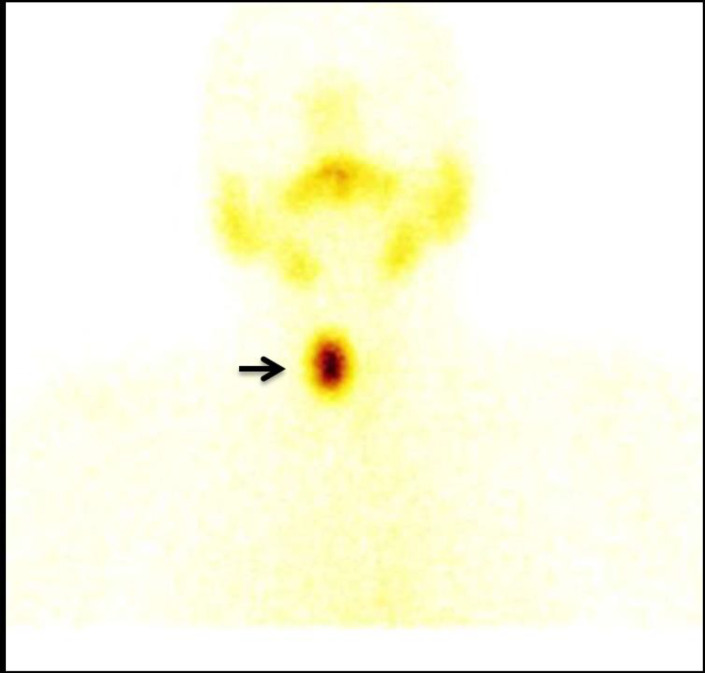
Anterior planar static images of the Tc-99m pertechnetate thyroid scan showing a hot nodule in the right lobe of the thyroid gland with suppressed tracer activity in the rest of the thyroid gland

 However, in view of the past history of hypothyroidism, the pre-operative thyroid profile was done, which showed a suppressed thyroid stimulating hormone (TSH) level of 0.01 μIU/ml (normal range: 0.35-5.5) with normal T_3_ and T_4_ levels. She had no clinical symptoms or signs suggestive of thyrotoxicosis. The patient underwent ^99m^Tc-pertechnetate thyroid scinti-graphy to evaluate for the cause of the suppressed TSH levels. The thyroid scan (Figure 2) performed 20 minutes after intravenous injection of 4 mCi (~148 MBq) of ^99m^Tc-pertechnetate revealed a “hot” nodule in the right lobe of the thyroid gland on the anterior planar static images of the neck, corresponding to the MIBI uptake. Tracer activity in the rest of the thyroid gland and background was suppressed and a diagnosis of functioning toxic thyroid adenoma was made. The patient was treated with radioactive iodine ablation (RAI) using 12 mCi (444 MBq) of oral I-131 sodium iodide solution instead of the planned surgery. Six months post RAI therapy, she presented with clinical and biochemical features of hypothyroidism (TSH – 14.9 uIU/ml) and was started on a replacement dose of LT_4 _(75 mcg). Following a further follow-up period of one year, she was found to be euthyroid (TSH-2.6 μIU/ml, T_3_-1.15 ng/ml and T_4_-9.5μg/dl) with the same replacement dose of LT_4_ and her biochemical levels also normalized (Calcium-9.4 mg/dl; Phosphate-3.5 mg/dl; iPTH-46.7pg/ml).

## Discussion

 In patients with hypercalcemia, routine evaluation includes an assessment of serum PTH levels to look for the presence of hyper-parathyroidism. USG neck and dual-phase Tc-99m sestamibi scintigraphy are used for pre-operative localization of PA (4). PA may sometimes occur in an intra-thyroidal location with an incidence of about 5%. PA is identified on dual-phase sestamibi parathyroid scintigraphy based on the relatively increased tracer uptake in the PA as compared to the thyroid gland and slower differential washout in delayed phase image (5, 6). Neoplastic thyroid nodules including malignancies are frequently known to show increased tracer uptake on the sestamibi scan (7). Large PAs must also raise the suspicion of a parathyroid carcinoma. However, positive sestamibi scan along with appearance as a ‘hot nodule’ on Tc-99m pertechnetate thyroid scan has been reported in case of functioning thyroid adenoma and in a case of colloid nodular goitre (8). On the other hand, slightly lower than 20% of patients with hyperthyroidism may be associated with asymptomatic hypercalcemia which may be PTH independent with enhanced bone resorption. Hyperthyroidism usually produces the condition of accelerated bone turnover which may lead to asymptomatic or symptomatic hypercalcemia depending upon the severity (2). Hence, a functioning thyroid adenoma producing hypercalcemia must be considered as one of the possible differential diagnoses while evaluating a case of hypercalcemia with suspected intra-thyroidal parathyroid adenoma. Evaluation of the thyroid profile followed by a Tc-99m pertechnetate thyroid scan can help in achieving the correct diagnosis. Treatment of toxic thyroid adenoma may control the hypercalcemia and features of hyperthyroidism, though the index patient did not have any clinical manifestations. 
